# What evidence exists on the impacts of large herbivores on climate change? A systematic map protocol

**DOI:** 10.1186/s13750-022-00270-2

**Published:** 2022-04-19

**Authors:** Jennifer Ramsay, Christopher Sandom, Thomas Ings, Helen C. Wheeler

**Affiliations:** 1 School of Life Sciences, Anglia Ruskin University, East Road, Cambridge, CB1 1PT UK; 2 School of Life Sciences and Sussex Sustainability Research Programme, University of Sussex, Brighton, BN1 9RH UK

**Keywords:** Albedo, Browsing, Carbon, Climate feedbacks, Grazing, Herbivory, Restoration, Rewilding Wildfire

## Abstract

**Background:**

In recent years there has been an increased focus on the role of large herbivores in ecosystem restoration and climate change mitigation. There are multiple processes by which large herbivores could potentially influence climate feedback and forcing effects, but the evidence has not yet been synthesised in a systematic and accessible format. Grazing, browsing, trampling, defecation, and seed dispersal by large herbivores can influence vegetation and soils in ways that may directly or indirectly contribute to climate change or mitigation. For example, changes in vegetation could impact wildfire regimes, carbon storage, and albedo, with ultimate impacts on climate. These processes may be influenced by herbivore species composition, density, and functional traits.

The main aim of this systematic map is to synthesise the range of research on climate feedback and forcing effects from large herbivores (≥ 10 kg) in terrestrial ecosystems. We also aim to identify knowledge clusters and gaps in the research base, as well as assessing the potential for quantitative analyses.

**Methods:**

A search of peer-reviewed and grey literature will be conducted using a range of bibliographic databases, search engines and websites. The search strategy will involve using a pre-defined search string with Boolean operators. All search results will be screened for relevance according to specific eligibility criteria. Screening will be conducted in two stages: all articles will initially be screened by title and abstract, then those that meet the eligibility criteria will be screened by full text. At both stages, articles will be excluded if they don’t meet all eligibility criteria or if they meet any exclusion criteria.

All articles included as eligible after full text screening will be coded. At each stage (of screening and coding) a proportion of articles will be processed independently by two reviewers to assess inter-reviewer reliability and resolve differences. The evidence will be presented in a searchable database with accompanying visual outputs. A narrative synthesis will be provided outlining the range and distribution of evidence, knowledge gaps and clusters, potential bias, and areas for further research.

**Supplementary Information:**

The online version contains supplementary material available at 10.1186/s13750-022-00270-2.

## Background

Many large herbivores are considered to be keystone species or ecosystem engineers, performing major ecological roles through their impacts on vegetation, nutrient cycling, and food webs [1, 10, 16]. In recent years there has been increasing recognition that by modifying ecosystems, large herbivores may exert a significant impact on climate feedback and forcing effects [9, 39].

### Climate effects

Climate effects that could potentially be modified by large herbivores include:

- 1. Land surface albedo: Grazing, browsing and trampling by large herbivores can alter vegetation structure and composition in ways that increase albedo, for example by reducing tree cover or increasing snow cover [7, 14, 22],te [43]. Changes in land surface albedo can lead to climate feedback effects, with lower albedo having warming effects and higher albedo having cooling effects [20].
- 2. Carbon storage and flux: Herbivore-induced changes in vegetation biomass and seed dispersal may also impact carbon storage and flux [4, 13, 15, 30]. For example, seed dispersal by large herbivores can increase carbon storage by increasing the recruitment of larger tree species [13]. Conversely, a reduction in woody vegetation through browsing could reduce carbon storage [4].
- 3. Wildfire regimes: Wildfires have multiple potential impacts on climate feedbacks [20]. Wildfire intensity and frequency could be altered by herbivore-induced changes in flammable woody vegetation or grass structure and composition [19, 38].
- 4. Methane emissions: Large herbivores may also influence climate through direct methane emissions as a by-product of digestion. These emissions vary according to species, digestive traits, and diet [37]. Methane emissions may also be influenced by herbivore impacts on vegetation, water levels and soil moisture and compaction.
- 5. Nutrient cycling: Large herbivores can have multiple indirect impacts on nutrient cycling [36]. Changes in nutrient cycles can influence plant growth (with consequences for carbon storage) and greenhouse gas fluxes, such as nitrous oxide, methane and carbon dioxide [24].

### Causal pathways and processes

**Fig. 1:**
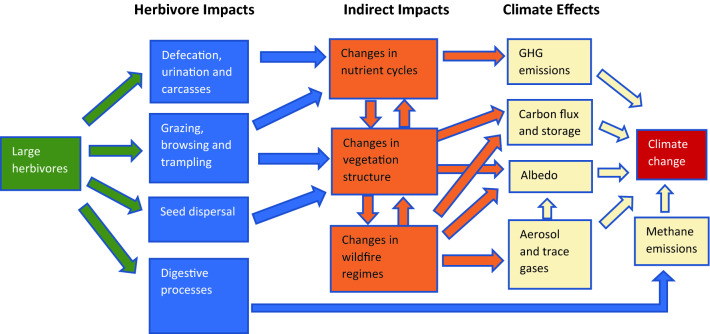
Simplified Conceptual Model. The introduction of large herbivores to an ecosystem may lead to multiple processes that ultimately influence climate change. Grazing, browsing and trampling can lead to changes in vegetation structure and nutrient cycles, which in turn could influence wildfire regimes and a variety of climate feedback and forcing effects. Climate could also be affected by direct methane emissions from herbivores, the impacts of defecation on nutrient cycling, and indirect impacts of seed dispersal on carbon storage

The impacts of large herbivores on vegetation dynamics and climate may vary significantly with species composition, density, habitat type, local climate, site history and management [2, 5]. There are multiple potential routes by which large herbivores could influence climate feedback and forcing effects (see Fig. 1). Due to the complexity of potential causal pathways and interactions between these pathways, it can be difficult to determine causes and effects from single studies. A synthesis of the evidence could help to elucidate these complex processes and their relative impacts on climate feedback and forcing effects.

### Purpose of the systematic map

Despite the significant role of large herbivores in nature restoration, there is currently no literature that draws together all relevant research to provide an overview of climate effects from large herbivores. This makes it difficult for practitioners and policy makers to identify the possible climate implications of decisions regarding land management with large herbivores. This systematic map aims to identify all relevant literature and synthesise the data in a format that is accessible and searchable for researchers, practitioners and decision makers. This will help to identify trends and clusters in the research, potential bias in the research base, and to highlight knowledge gaps where further research is needed. It will identify specific areas of research where more detailed qualitative and quantitative analyses may be feasible for future research.

### Stakeholder engagement

To enhance the relevance of the systematic map to practitioners and policy makers, a range of key stakeholder groups will be consulted through workshops and meetings. An advisory panel will also be established consisting of four stakeholder representatives. The main purpose of the panel is to contribute a range of expert and practitioner perspectives, which will improve the relevance of the systematic map to end users. The panel will also discuss policy and land management implications and agree priority areas for further research. One-off workshops and meetings will also be held with a wide range of stakeholders, including representatives of land managers, farming communities, nature conservation bodies, rewilding groups, and climate experts.

Initial discussions of the systematic map concepts and Protocol have been carried out with three stakeholder groups (representatives of Rewilding Europe, Rewilding Britain, and the Endangered Landscape Programme). All three independently identified the need for clearer evidence of the climate impacts of large herbivores, and for the evidence to be presented in a meaningful and accessible way for practitioners, decision makers and funding bodies. All three identified the need for information on how climate impacts may vary for different herbivore species, at different densities, and in different habitats.

## Objective of the systematic map

### Objective

The main objective of the systematic map is to identify and collate research relating to climate feedback and forcing effects from large herbivores. All relevant research will be coded and analysed to identify research clusters, trends and knowledge gaps. This will provide a detailed overview of the existing state of knowledge and gaps in the research.

### Primary question

What evidence exists on climate feedback and forcing effects from large herbivores?

### Sub-questions

What evidence exists on large herbivore modification of climate through the following mechanisms: land surface albedo; wildfire regimes; carbon storage and flux; methane emissions; nutrient cycles?

What are the knowledge gaps, clusters and potential biases in the research base? For example, are there research clusters or gaps for particular species, biomes, climate effects or study types? Is there more focus on processes or states as outcomes? Is there any evidence of geographic bias?

### Definitions of the questions:

The key PECO elements of the questions are:

- Population: All terrestrial habitats.
- Exposure: Introduction of large herbivores (or change in density or species composition of large herbivores).
- Comparator: No large herbivores or difference in density or species composition of large herbivores.
- Outcome: Changes in climate feedback and forcing effects (eg. albedo, carbon storage, carbon flux, wildfire regimes, methane emissions, nutrient cycles).

## Methods

The methods will follow the *Guidelines and Standards for Evidence Synthesis in Environmental Management* [8] and the ROSES reporting standards for Systematic Map Protocols [18] (See Additional File 3).

### Search strategy

#### Bibliographic databases

A search of five bibliographic databases will be conducted (see list below). These databases have been selected based on their relevance to the field of study and their comprehensiveness.

Bibliographic databases to be searched:

- Web of Science: Core Collection
- Scopus
- Science Direct
- GeoRef
- JSTOR

#### Search engines

Two web-based search engines (Google Scholar and Microsoft Academic) will be searched to identify academic or grey literature not captured by the search of bibliographic databases.

#### Websites

Fifteen organisational and governmental websites will be searched to identify relevant grey literature or other documents not identified through bibliographic databases. Due to the restriction of searches to English language, this could produce geographical bias in the website search results. This will be reduced by contacting relevant organisations with a global or continental reach (such as the Global Rewilding Alliance and Rewilding Europe), who include member organisations from non-English speaking countries.

Websites to be searched:

- Rewilding Europe
- Rewilding Britain
- Global Rewilding Alliance
- GrazeLIFE
- RSPB
- Wildlife Trusts
- Natural England
- NatureScot
- Natural Resources Wales
- United Nations Environment Programme
- European Commission Joint Research Centre
- European Environment Agency
- GRID Arendal
- International Union for Conservation of Nature
- United Nations Environment Programme

#### Stakeholder contacts and authors

Stakeholders, relevant organisations, and authors of key articles will be contacted by email or in person to enquire if they are aware of additional unpublished research.

### Search string scoping

Searching of bibliographic databases will be conducted using a search string. The search string has been tested and optimised by conducting a scoping exercise in Web of Science, following the CEE guidelines [8]. Narrower and wider search strings were trialled during scoping to ensure an appropriate balance of specificity (reducing the number of irrelevant studies) and sensitivity (ensuring all relevant studies are identified).

Nine different iterations of the search string were trialled. The specificity of each trial search string was assessed by modifying terms to see how many documents were returned by Web of Science in the ‘Topic’ field (which includes Title, Abstract and Keywords). The comprehensiveness of each search string was tested by listing 20 relevant articles already known to the authors (Additional file 1) then checking if these articles were returned by the search string in Web of Science. These articles were chosen due to their relevance to the topic and the breadth of relevant research covered by these articles, including herbivore impacts on albedo, wildfire regimes, carbon storage, nitrogen cycles and methane emissions.

The widest search strings with more terms were found to return too many non-relevant articles (> 50,000), while the narrowest search strings did not return the full 20 articles of known relevance. Of the search strings trialled, the search string below was found to be the optimum for specificity and sensitivity, returning all 20 of the test articles, and a total of 33,094 articles (search date 17/11/2021).

### Search string (Web of Science Format)

(Herbiv* OR Graz* OR Brows* OR Rewild* OR Exclos*).

AND

(Climat* OR Albedo OR Fire OR Wildfire OR Carbon OR Methane OR Greenhouse OR Global OR ‘Nutrient Cycl*’).

The search string uses the Boolean operators OR and AND to identify literature that includes both herbivory-related terms and climate-related terms. Within each bibliographic database, the search string will be adapted to the format required for that database but will use the same terms and search fields (Title, Abstract and Keywords).

### Website and search engine searches

Search Engines: Google Scholar and Microsoft Academic will be searched to identify grey literature that may not have appeared in the bibliographic databases. Due to the limitations of using search engines for systematic reviews, we will follow the recommendations of Haddaway et al. [17], including searching by Title only and downloading only the first 300 search results (ordered by relevance) for inclusion in the screening process.

Websites: As most organisational websites don’t provide for Boolean operators, each website will be searched with the following key terms:

- Herbivores and climate
- Herbivores and wildfire
- Herbivores and albedo
- Herbivores and carbon
- Herbivores and nutrient cycles
- Herbivores and methane

Only English language searches will be conducted due to limited resources of the research team. The impact of our focus on English language literature will inform our interpretation of any geographic biases identified by the Systematic Map. The search will be updated if original searches were conducted more than two years prior to review completion. Any additional papers identified will be added to the Systematic Map.

## Article screening and study eligibility criteria

### Screening strategy

Two stages of screening will be conducted. The first stage will be Title and Abstract screening where the relevance of each article will be assessed by title and abstract. Articles that clearly meet the exclusion criteria will be excluded. Articles that meet the eligibility criteria (or where there is uncertainty) will be included for screening at stage two. Stage two will involve Full Text screening, where each article assessed to be relevant at the first stage will be screened on the basis of full text. Articles that meet any of the exclusion criteria will be excluded. Articles that meet all of the eligibility criteria will be included. Where there is uncertainty, articles will be included and marked for a second opinion.

Screening will be conducted using drop-down menus listing exclusion criteria. This will allow the reasons for exclusion to be recorded for each article. This process will be conducted using specialist software for systematic mapping and reviews (EPPI-Reviewer Web [44]). This software will also be used to remove duplicate articles when the same article is returned by more than one bibliographic database or search engine. Due to the large number of articles to be screened, and the likely high proportion of non-relevant articles, search results will be ordered by relevance in the bibliographic database searches and search engines. Articles retrieved from the searches will be imported to EPPI-Reviewer in batches of 1000 (the WoS export file limit) ordered by relevance. The batches will be screened in relevance order to ensure that batches containing the most relevant articles are screened first. The first stage of screening will be stopped when more than 50% of articles have been screened *and* 500 non-relevant articles occur in a row. The remaining batches will be checked by random screening of 10% of articles from each batch.

### Inter-reviewer reliability

At each stage of the screening process, a proportion of articles will be screened by two reviewers to assess consistency of decisions between reviewers. At title and abstract screening, 500 articles will be randomly selected to be screened by a second reviewer. At full text screening, 10% of articles will be screened by a second reviewer.

At each stage a Cohen’s kappa test [6, 31] will be conducted to assess the degree of agreement between the two reviewers (inter-rater reliability). If the kappa result is over 0.6, this will be considered an acceptable level of agreement for inter-reviewer reliability and any disagreements will be discussed and resolved. If the kappa result is less than 0.6 the eligibility and exclusion criteria will be discussed (and amended if necessary) by the review team to improve consistency of interpretation between reviewers. The process will be repeated at each stage until a kappa result over 0.6 is achieved.

Procedural independence: Any member of the review team who is listed as an author on an article will not be involved in any decisions relating to that article.

### Eligibility criteria

All articles will be included or excluded at screening according to the following PECO criteria:

• Population: All terrestrial habitats.
• All terrestrial habitats will be included. Habitats that are exclusively aquatic will be excluded. Terrestrial wetland habitats (such as marsh, bog and fen) will be included.
• To produce a broad and globally relevant systematic map, research conducted in all geographical locations will be included.
  Exposure: Introduction of large herbivores or change in density or species composition.
• As the systematic map concerns the impacts of *large* herbivores, studies will be excluded if they relate only to herbivore species smaller than 10 kg in adult weight. There are a variety of definitions of ‘large herbivore’ in the literature. Owen-Smith [35] defines three broad categories of ‘large herbivore’: Megaherbivores (over 1000 kg),Macroherbivores (100–1000 kg); and Mesoherbivores (10–100 kg). For the purposes of this study, we are using the 10 kg threshold as this will allow the inclusion of most goats and small deer (which may be of importance in management decisions), whilst excluding rodents, lagomorphs and other small vertebrates to ensure a manageable timeframe and focus for the systematic map. We will extract body mass data from peer-reviewed literature and databases, including panTHERIA [21], HerbiTraits [28] and Body Mass of Late Quaternary Mammals [40]. Where possible, the average of male and female body mass will be used, following the methods of Smith et al. [40].
• All taxonomic groups will be included. Although the majority of research has focused on ungulates, we are including all taxonomic groups (such as primates, ratites, testudines and marsupials) to provide an overview of evidence for less-studied groups. The coding process will allow the evidence for different taxonomic groups to be differentiated.
• As the systematic map relates to terrestrial herbivores, studies that involve exclusively aquatic herbivores will be excluded from the systematic map. Semi-aquatic herbivores (such as beavers and capybara) will be included when the impact studied is on terrestrial habitats.
• Studies that involve simulation or modelling of impacts by large herbivores will be included.
• Studies that involve the introduction or reintroduction of large herbivores, or a change in species or density of large herbivores (including exclosures) will be included.
• Studies will be included if they involve any type of impact by large herbivores (eg. browsing, grazing, trampling, defecation etc.) and its effects on climate feedback or forcing effects.
  Comparator: No large herbivores or difference in density or species composition of herbivores.
• We will include all studies where the comparator is a change in the presence/absence of large herbivores, or a change in density or species composition of large herbivores.
• We will also include studies where the comparator is a difference in management or habitat variables of large herbivores, or where herbivore impacts are compared to other interventions (eg. mowing, burning).
• Studies involving simulation or modelling of herbivory as the comparator will also be included.
  Outcome: Changes in climate feedback or forcing effects (eg. albedo, carbon storage, carbon flux, wildfire regimes, methane emissions, nutrient cycles).
• We will include all studies that address the impact of large herbivores on any aspect of climate feedback or forcing effects.
• The search string is likely to return studies of the impacts of climate change on herbivores. As the systematic map concerns the impacts of large herbivores on climate (not vice-versa), studies will be excluded if they relate only to the impacts of climate change or climatic variables on herbivores.

### Other eligibility criteria

- Study type: All study types that include original data will be included (eg. observational, remote sensing, experimental, modelling etc.) to produce as broad a systematic map as possible.
- Theoretical papers: Papers that are purely theoretical will be excluded from the coding process but will be listed separately and referred to in the discussion.
- Review papers: Review papers will be excluded from the coding process, but individual studies referred to in the review will be identified and included or excluded separately in the screening process.
- We will exclude papers that report on data already reported elsewhere. This will be done by cross-checking references and citations.

Excluded articles: All articles excluded at full text will be listed and reasons for exclusion provided.

### Study validity assessment

As the systematic map is intended to provide a broad overview of research, individual studies will not be critically appraised but study design will be coded.

### Data coding strategy

For each eligible study that passes the screening at full text stage, meta-data and relevant variables will be extracted and coded as shown in Additional file 2: Table S1. The herbivore-related coding terms are adapted from the coding used by Soininen et al. [41]. We will use EPPI-Reviewer Web software [44] to facilitate coding with drop-down menus and to ensure consistency of coding between reviewers.

The coding strategy will be piloted by two independent reviewers on a sub-set of fifteen full-text articles each. Ten of these articles will be identical to check for reviewer consistency. Any inconsistencies will be discussed and, if necessary, coding variables will be amended to improve consistency. The pilot will be used to assess the suitability and comprehensiveness of the coding variables, as well as the repeatability of the process.

In articles that contain more than one original evidence point (for example multiple research questions, studies or data sets), each original evidence point will be recorded separately with a unique ID. Where relevant data is unclear or missing from studies, attempts will be made to contact the authors.

## Study mapping and presentation

The systematic map will provide a narrative synthesis, searchable database and visual outputs, describing and mapping the evidence base for the impacts of large herbivores on climate feedback effects. The full data extracted from each study will be made available in a database, allowing users to filter, search and sort the evidence. Statistics, tables and charts will be presented to summarise, group and visualise the evidence. Interactive evidence maps will be generated to reveal knowledge gaps and clusters in the research using EPPI-Mapper software [12]. This will allow identification and discussion of areas for further research, as well as revealing under-studied species, geographical locations or biomes.

The evidence will be analysed and discussed in relation to knowledge clusters, which could form topics for more detailed systematic reviews or quantitative analyses. For example, there may be knowledge clusters around particular herbivore species, habitats, or climate feedback effects that could allow for more detailed analyses and critical appraisal of the research base. The outcomes of the systematic map will be discussed with key stakeholders in relation to policy and practice, including implications for land management, ecosystem restoration and climate policy. Policy and management implications will be outlined and discussed in the narrative synthesis.

## Supplementary Information

**Additional file 1. ** Articles used in Search String Scoping.

**Additional file 2:** **Table S1.** Coding Variables.

**Additional file 3:** ROSES form for Systematic Map Protocol.

## Data Availability

Not applicable.
