# Supplementary material for: What evidence exists on the impacts of large herbivores on climate change? A systematic map protocol
Source: Environ Evid. 2022 Apr 19;11:14. doi: 10.1186/s13750-022-00270-2 (PMC11378845; doi:10.1186/s13750-022-00270-2)
Supplement: Supplementary file 1 — Additional file 1. Articles used in Search String Scoping. [file 13750_2022_270_MOESM1_ESM.docx]

**Appendix A**

Papers of known relevance selected for search string scoping (see References for full paper details):

1. (Bakker and Svenning, 2018)
2. (Cahoon et al., 2012)
3. (Cohen et al., 2013)
4. (Cromsigt et al., 2018)
5. (Dangal et al., 2017)
6. (Forbes et al., 2019)
7. (Kolstad et al., 2018)
8. (Laiho et al., 2017)
9. (Leroux et al., 2020)
10. (Liu et al., 2020)
11. (Macias-Fauria et al., 2020)
12. (McSherry and Ritchie, 2013)
13. (Olofsson et al., 2001)
14. (Olofsson, 2009)
15. (Rouet-Leduc et al., 2021)
16. (Sandom et al., 2020)
17. (Tanentzap and Coomes, 2012)
18. (te Beest et al., 2016)
19. (Viglizzo et al., 2019)
20. (Ylänne et al., 2020)
