# Supplementary material for: What evidence exists on the impacts of large herbivores on climate change? A systematic map protocol
Source: Environ Evid. 2022 Apr 19;11:14. doi: 10.1186/s13750-022-00270-2 (PMC11378845; doi:10.1186/s13750-022-00270-2)
Supplement: Supplementary file 2 — Additional file 2: Table S1. Coding Variables. [file 13750_2022_270_MOESM2_ESM.docx]

**Appendix B**

**Table S1: Coding Variables:** Coding variables and descriptions to be used in the data extraction and coding process (adapted from the coding format in Soinenan et al., 2018). In the ‘Source’ column, P indicates the data will be extracted directly from the publication; C indicates the data will be extrapolated by the authors when it is not directly stated in the publication.

| **Topic** | **Coding variable** | **Variable description** | **Comments** | **Source** |
| --- | --- | --- | --- | --- |
| Publication | Authors | List of authors |  | P |
|  | Title | Title of article |  | P |
|  | Journal | Journal or publishing house |  | P |
|  | Year | Year of publication |  | P |
|  | Language | Language |  | P |
|  | Study point ID | Study point ID | An individual ID will be allocated for separate study points in the same paper. | C |
| Study location | Country | Country of study | Country of study will be coded and continent or global region added to database post-coding. | P |
|  | Latitude | Latitude of study location (or other geographical coordinates as published) |  | P |
|  | Longitude | Longitude of study location (or other geographical coordinates as published) |  | P |
|  | Elevation | Elevation | Reported elevation will be recorded then assigned a scale (1-5) post-coding. | P |
| Study details | Study_type | Type of study eg. experimental, observation, modelling etc. |  | P/C |
|  | Experimental_design | Experimental design eg. Before-After (BA), Control-Impact (CI), After (A), Before-After-Control-Impact (BACI) |  | P/C |
|  | Study_method | Study method eg. remote sensing, field study etc. |  | P/C |
|  | Exposure | Method of quantifying herbivore exposure eg. exclosure, gradient of herbivore density, space-for-time substitution |  | P/C |
|  | Other_exposures | Any other exposures that herbivory was compared with eg. disturbance events, mowing, fire etc. |  | P |
|  | Spatial_area | Size of study area | Reported size of study area will be recorded, then assigned a scale (1-5) post-coding. | P/C |
|  | Study_length | Length of study | Reported length of study will be recorded, then assigned a scale (1-5) post-coding. | P/C |
|  | Temporal_resolution | Interval and regularity between measurements | Measurement interval will be recorded, then assigned a scale (1-5) post-coding. | P/C |
|  | Process_state | Does study address dynamic ecological processes or fixed state? |  | C |
|  | Redundancy | Is data already reported in another study? |  | C |
| Population: habitat or land area | Habitat_type | Habitat type(s) as reported |  | P |
|  | Current_land_use | Current land use type(s) |  | P |
|  | Previous_land_use | Previous land use type(s) |  | P |
|  | Biome_type | Biome type |  | P |
|  | Vegetation_type | Dominant vegetation of study area as reported |  | P |
|  | Conservation_status | Conservation status of study location eg. protected area |  | P |
|  | Manage_habitat | Current and previous habitat management eg. mowing, burning, scrub removal etc. |  | P |
| Exposure: herbivory | Herbivore_species | Species of large herbivore involved in the study | The species name(s) will be recorded in the coding. Functional traits and taxonomic group classifications for each species will be added to the database post-coding. Species richness of large herbivores will also be added post-coding. | P/C |
|  | Herbivory_season | Season when herbivory occurs |  | P |
|  | Effect_on_plants | Impact of herbivores on plants species eg. removal of plant parts, trampling, seed dispersal |  | P |
|  | Manage_herbivore | Management of herbivore eg. supplementary feeding, culling, hunting, food production, farming |  | P |
|  | Introduced_status | Introduced status of herbivore eg. native, reintroduced, introduced, naturally recolonised etc. |  | P |
|  | Density_herbivore | Density of each herbivore species pre-intervention | Reported density will be recorded then assigned a scale post-coding. | P |
| Comparator | Herbiv_species_pre | Species of large herbivore prior to intervention | The species name will be recorded in the coding. Functional traits and taxonomic group classifications for each species will be added to the database post-coding. | P |
|  | Herbiv_season_pre | Season when herbivory occurred prior to intervention |  | P |
|  | Effect_plants_pre | Impact of herbivores on plants species prior to intervention eg. removal of plant parts, trampling, seed dispersal |  | P |
|  | Manage_herb_pre | Management of herbivore eg. supplementary feeding, culling, hunting, food production, farming |  | P |
|  | Introduced_herb_pre | Introduced status of herbivore eg. native, reintroduced, introduced, naturally recolonised etc. |  | P |
|  | Density_herb_pre | Density of each herbivore species pre-intervention | Reported density will be recorded then assigned a scale post-coding. | P |
| Outcome | Climate_effect | Type of climate feedback or forcing effect eg. albedo, wildfire, soil carbon, above- or below-ground carbon, carbon flux, methane emissions etc. |  | P |
|  | Direction_effect | No change, increase or decrease in climate feedback or forcing effect |  | P |
|  | Magnitude_effect | Magnitude of change in climate feedback or forcing effect | Magnitude of change will be recorded as reported. This will be assessed post-coding for potential further analyses. | P |
| Modifier (ecological context) | Temperature | Temperature (degrees Celsius) |  | P |
|  | Temperat_measure | Measurement of temperature eg. mean annual temperature, temperature of warmest/coldest months |  | P |
|  | Precipitation | Precipitation (mm) |  | P |
|  | Precipitat_measure | Measurement of precipitation eg. annual or seasonal, mean or sum |  | P |
|  | Growing_season | Length of growing season |  | P |
|  | Permafrost | Presence or absence of permafrost |  | P |
|  | Disturbance | Disturbance events occurring in the habitat e.g. storm, fire, drought, flood |  | P |
|  | Predators | Any predators recorded by the authors |  | P |
|  | Other_herbivores | Other herbivores recorded by the authors |  | P |
|  | Productivity | Productivity of habitat as described by the authors |  | P |
|  | Bioclimatic_zone | Bioclimatic zone |  | P |
|  | Soil_type | Soil type |  | P |
